# Size-Dependent Ultrasonic Characterization of Cylindrical Surrogate Targets Using Through-Transmission Ultrasound

**DOI:** 10.3390/bioengineering13101122

**Published:** 2026-09-26

**Authors:** Gongmin Rim, Zhongsoo Lim, Kwanyong Hyun

**Affiliations:** 1 Department of Thoracic and Cardiovascular Surgery, CHA Bundang Medical Center, CHA University School of Medicine, Seongnam 13496, Republic of Korea; yim8585@chamc.co.kr; 2 TFYHNN., Pohang 37660, Republic of Korea; zslkr@yahoo.com; 3 Department of Thoracic and Cardiovascular Surgery, St. Vincent’s Hospital, College of Medicine, The Catholic University of Korea, Seoul 06591, Republic of Korea

**Keywords:** extracorporeal membrane oxygenation, through-transmission ultrasound, cylindrical surrogate target, detection index, signal processing

## Abstract

Background: Thrombus formation remains a major complication during extracorporeal membrane oxygenation (ECMO), potentially resulting in circuit failure and thromboembolic events. Although several techniques have been proposed for thrombus detection, the relationship between measured ultrasonic features and target size has not been systematically characterized. This study evaluated size-dependent ultrasonic signal changes using standardized cylindrical surrogate targets under controlled conditions. Methods: A through-transmission ultrasonic system incorporating paired point-focused transducers (5 and 10 MHz), a water chamber, an ultrasonic pulser/receiver, a digital storage oscilloscope, and a manual translation stage was constructed. Cylindrical targets made of acrylic, acrylonitrile butadiene styrene (ABS), and SUS304 stainless steel, with diameters ranging from 0.1 to 5.0 mm, were scanned at 0.5 mm intervals over a 30 mm range. Baseline-subtracted waveforms were used to calculate peak height, pulse area, and squared-amplitude sum. Results: Exploratory regression analyses demonstrated positive diameter-dependent associations for all evaluated features. Pulse area showed strong linear associations across the six material–frequency conditions (R^2^ = 0.904–0.997), although no single feature consistently demonstrated the highest goodness of fit. Strong associations were observed at both frequencies; however, the different receiver gains precluded direct inference regarding relative frequency sensitivity. Under the 10 MHz condition, the 0.3 mm SUS304 wire target was the smallest tested surrogate target meeting the study-specific operational detection-index criterion (DI > 3). Conclusions: Through-transmission ultrasound demonstrated size-dependent changes in signals obtained from standardized cylindrical surrogate targets. Pulse area was identified as a practical candidate feature because of its consistently strong associations and computational simplicity, but neither statistical superiority nor a predictive sizing model was established. The 0.3 mm result applies to a high-acoustic-contrast SUS304 target under the specified experimental conditions and does not represent a biological thrombus detection limit.

## 1. Introduction

Extracorporeal membrane oxygenation (ECMO) has become an indispensable life-support modality for patients with severe cardiac or respiratory failure. Despite continuous advances in ECMO technology and anticoagulation strategies, thrombus formation within the ECMO circuit remains one of the major complications affecting both circuit durability and patient safety [1,2,3,4,5]. Thrombi generated within the extracorporeal circuit may impair oxygenator performance and, more importantly, detach and embolize into the systemic circulation, potentially causing stroke, peripheral embolism, or other life-threatening complications [6,7,8]. Therefore, continuous monitoring of thrombus formation, particularly at locations immediately upstream of the patient, is essential for preventing thromboembolic events.

Several techniques have been proposed for thrombus monitoring in ECMO circuits, including pressure-gradient monitoring, optical sensors, hyperspectral imaging, infrasound, and laboratory coagulation markers [6,9,10,11,12,13,14,15]. Although these methods have demonstrated varying degrees of feasibility, most are designed to detect thrombus formation within oxygenators or circuit components rather than individual thrombi traveling through the bloodline. Furthermore, currently available methods primarily provide qualitative or indirect information and do not allow continuous real-time characterization of size-dependent properties of individual circulating thrombi, which may provide clinically relevant information beyond thrombus detection alone [6,15].

Ultrasonic techniques have long been used for material characterization because acoustic wave propagation is influenced by the density, elasticity, and acoustic impedance of the target medium. Through-transmission ultrasound has also been applied to detect changes in material structure in nonbiological systems [16], illustrating the sensitivity of transmitted ultrasonic signals to changes in the acoustic pathway. During blood coagulation, fibrin formation and changes in red blood cell aggregation alter ultrasonic attenuation, backscatter, and propagation velocity, enabling noninvasive monitoring of clot development [17,18,19,20]. These acoustic changes occur alongside evolving fibrin-network structure and mechanical properties during clot formation [21,22]. Previous investigations have demonstrated that ultrasonic parameters can detect blood coagulation and monitor thrombus formation in vitro; however, relatively few studies have focused on detecting and characterizing individual thrombi under flowing conditions similar to those encountered in ECMO circuits.

In our previous study, we developed a noninvasive ultrasonic sensing system capable of detecting individual thrombi flowing through an ECMO bloodline by measuring transient changes in ultrasonic propagation time [23]. The study demonstrated the feasibility of real-time thrombus detection under various ECMO flow conditions and established ultrasound as a promising monitoring modality for extracorporeal circulation.

Nevertheless, the relationship between target dimensions and through-transmission ultrasonic signal characteristics has not been systematically characterized. Characterizing this relationship is an important prerequisite for the future development of quantitative thrombus-sizing methods. Therefore, the present study investigated size-dependent changes in through-transmission ultrasonic signal features using standardized cylindrical surrogate targets under controlled experimental conditions.

## 2. Materials and Methods

The experimental procedures were based on our previously reported ultrasonic thrombus detection system with minor modifications for size-dependent surrogate target characterization experiments [23].

### 2.1. Experimental Setup

An ultrasonic thrombus monitoring system was constructed using a water chamber as a controlled acoustic medium for the surrogate target measurements. The system consisted of two custom-built focused ultrasonic transducers (USP Co., Daejeon, Republic of Korea) with center frequencies of either 5 or 10 MHz, a diameter of 1/4 inch, and a focal length of 2 inches. The two frequencies were evaluated to compare size-dependent ultrasonic responses at different operating frequencies under the same experimental conditions. Ultrasonic pulses were generated and received using a JSR DPR35+ pulser/receiver (JSR Ultrasonics, Pittsford, NY, USA), and the signals were digitized using a Siglent SDS1104X-E digital storage oscilloscope (Siglent Technologies, Shenzhen, China). The transducers were mounted on a manual three-axis positioning stage (SS3H-100, ST1 Co., Incheon, Republic of Korea) to enable precise alignment during measurements. The experimental setup is presented in Figure 1. Figure 1a shows the experimental water chamber, Figure 1b illustrates the ultrasonic measurement principle, and Figure 1c shows the cylindrical surrogate target mounted on the manual translation stage used for the sizing experiments. The overall experimental workflow, including target preparation, through-transmission ultrasonic scanning, RF signal acquisition, baseline removal, signal-feature extraction, and size-dependent characterization, is summarized in Figure 2.

### 2.2. Cylindrical Surrogate Targets

To characterize size-dependent ultrasonic responses and operational detectability using the proposed ultrasonic method [23], cylindrical surrogate targets with various diameters were prepared. Because fabricating stable thrombi with precisely controlled sizes is technically difficult, cylindrical targets made of acrylic, acrylonitrile butadiene styrene (ABS), and SUS304 stainless steel were used as surrogate materials. Acrylic and ABS were selected as standardized surrogate materials with acoustic impedances closer to those of biological thrombi than SUS304 stainless steel; however, commercially available specimens with precisely controlled submillimeter diameters were limited. SUS304 wires were therefore additionally used because of their commercial availability in smaller, precisely controlled diameters, allowing evaluation of the lower limit of size-dependent detectability despite their substantially different acoustic properties. Because their acoustic properties differ from those of biological thrombi, the present experiments were intended to characterize size-dependent ultrasonic responses rather than to directly reproduce the acoustic behavior of real thrombi. Representative literature values further illustrate these differences: acoustic impedances of approximately 3.2 MRayl for PMMA, 2.13 MRayl for ABS, and 42.79 MRayl for SUS304 stainless steel have been reported, whereas those of human clot, blood, and water are approximately 1.68, 1.72, and 1.48 MRayl, respectively, under previously reported experimental conditions [20,24,25,26]. The dimensions and materials of the cylindrical surrogate targets used in this study are summarized in Table 1. Each cylindrical target was mounted vertically on a manual translation stage and scanned horizontally through the ultrasonic focal region under controlled conditions, as shown in Figure 1c. All cylindrical targets had a length of at least 50 mm. Because the point-focused ultrasonic beam diameter was less than 2 mm in the target region, the target length was substantially greater than the insonified region. The central portion of each target was positioned within the focal region, sufficiently distant from both ends. Consequently, target-length and end effects were expected to be negligible under the present experimental configuration.

### 2.3. Ultrasonic Scanning Protocol

Each cylindrical surrogate target was mounted vertically on the manual translation stage, with its longitudinal axis aligned perpendicular to the ultrasound propagation direction within an angular error of 1°. The central portion of the target was positioned within the focal region and scanned horizontally across the point-focused ultrasonic beam. Scanning a 1 mm-diameter cylindrical target yielded an effective lateral interaction width of less than 2 mm. This measurement was used to estimate the target-affected scan region and did not constitute independent acoustic beam profiling. Initial target positioning and acquisition-window alignment were performed using the raw RF waveform. The total acquisition window was approximately 2.5 μs. Whenever the target was changed, the temporal position of the acquisition window was manually adjusted so that the first crossing of the 50 mV threshold in the raw RF waveform occurred at approximately 40% of the total window. This provided approximately 1.0 μs of pre-trigger recording and 1.5 μs of post-trigger recording. After this initial adjustment, the acquisition-window position was maintained unchanged throughout the complete 61-position scan of that target. The same holder, alignment procedure, scan direction, and positioning protocol were used for all target materials and diameters. The target was scanned horizontally across the ultrasonic beam. Measurements were performed at 0.5 mm intervals over a total scan distance of 30 mm, yielding 61 distinct spatial measurement positions within a single scan.

Ultrasonic RF waveforms were sampled at 500 MS/s. The pulser/receiver was operated at a pulse repetition frequency of 1 kHz with no damping, a 1 MHz high-pass filter, and no low-pass filter. Receiver gain was set to 47 dB for the 5 MHz measurements and 40 dB for the 10 MHz measurements. For the 10 MHz submillimeter detectability measurements reported in Table 2, the pulser output was 215 V. At each spatial position, 256 consecutive acquisitions were internally averaged by the digital storage oscilloscope to generate one stored RF waveform. The individual acquisitions were not separately stored or analyzed as independent measurements.

The same experimental procedure was performed for all target materials and diameters using both 5 MHz and 10 MHz transducers.

### 2.4. Signal Processing and Feature Extraction

The received ultrasonic RF waveforms were processed using spatial-reference baseline subtraction to isolate signal changes associated with the cylindrical surrogate targets. Each target was positioned at the center of the 30 mm scan range. Because the effective lateral interaction width estimated from the 1 mm target scan was less than 2 mm, the central approximately 10 mm region was conservatively regarded as potentially affected by the target and excluded from baseline calculation. Raw RF waveforms acquired over approximately 10 mm on each side of this central region (approximately 20 mm in total) were averaged sample by sample to generate the reference baseline waveform (RB). A separate reference baseline was generated for each target and transducer-frequency condition. Twenty scan positions on each side, corresponding to 40 baseline positions in total, were used to calculate each reference baseline waveform. Baseline stability was assessed by visually comparing the waveforms obtained from the two flanking baseline regions; no evident drift was observed. These flanking regions were selected because they were located well outside the estimated target-affected region and produced symmetric, visually stable waveforms without an evident target-associated response.

The difference waveform at each scan position was calculated as

*D_i_* = *R_i_* − *RB_i_*

where *R_i_* is the measured raw RF-signal amplitude and *RB_i_* is the corresponding reference baseline amplitude at sample *i*.

The raw RF waveforms were acquired using the target-specific acquisition window aligned as described in Section 2.3.

Following baseline subtraction, the pre-pulse background region (R1) and pulse-containing region (R2) were identified from the difference waveform. The first point at which the absolute difference-signal amplitude exceeded 50 mV was used as the boundary between R1 and R2. R1 extended from the beginning of the acquisition window to the sample immediately preceding this threshold crossing, whereas R2 extended from the first threshold crossing to the end of the acquisition window. The 50 mV threshold was used only for acquisition-window alignment and temporal-region selection; it was not used as the target-detection criterion. The same region selection procedure was applied to all target diameters.

Three quantitative features were extracted from R2: peak height (PH), pulse area (PA), and squared-amplitude sum (P). Because the difference waveform could contain either positive or negative deviations, peak height was defined as the maximum absolute difference-signal amplitude in R2:

$$PH=\underset{i\in R2}{\max}\left|D_{i}\right|$$

Pulse area was calculated as

$$\mathrm{PA}=\sum_{i=1}^{N}|D_{i}|$$

and the squared-amplitude sum was calculated as

$$P=\sum_{i=1}^{N}D_{i}^{2}$$

where D_i_ denotes the difference-signal amplitude of the i-th sample and N is the number of samples in R2. These features were subsequently analyzed as functions of measured target diameter. The R1 background data and R2 peak height were additionally used for the submillimeter-target detectability analysis described in Section 2.6.

These three features were selected because they describe complementary aspects of the target-induced waveform change. Peak height represents the largest instantaneous signal deviation and is therefore sensitive to the strongest local waveform change, but may become less responsive when the maximum amplitude approaches saturation. Pulse area integrates the absolute signal deviation over the complete pulse window and therefore reflects both amplitude and temporal extent without cancelation between positive and negative components. The squared-amplitude sum preferentially weights large amplitude deviations and provides an energy-related descriptor, although it is more strongly influenced by large individual samples and is computationally more intensive than pulse area.

For each target scan, peak height, pulse area, and squared-amplitude sum were first calculated independently at all 61 spatial positions. The maximum value of each resulting spatial feature profile was then selected as the representative target-associated response for that target diameter and was used in the diameter-dependent regression analysis. This procedure allowed for small differences between the nominal scan center and the spatial position of maximum target–beam interaction.

### 2.5. Statistical Analysis

Exploratory statistical analyses were performed separately for each target material, transducer frequency, and signal feature. Measured target diameter was treated as the independent variable, whereas peak height, pulse area, and squared-amplitude sum were analyzed separately as dependent variables. A simple linear regression model,

*y* = *β*_0_ + *β*_1_ d + *ε*

was fitted to each dataset, where d is the measured target diameter, β_0_ is the intercept, and β_1_ is the regression slope. The slope with its 95% confidence interval, coefficient of determination (R^2^), and corresponding two-sided *p*-value were reported. Spearman’s rank correlation coefficient (ρ) was additionally calculated to assess monotonic association without requiring linearity.

Because only a single measured value was available at each diameter, the analyses were considered exploratory. The confidence intervals describe uncertainty in the fitted regression coefficients and do not represent within-diameter measurement variability or repeatability. No definitive predictive sizing model was developed or validated in the present study.

Because receiver gain differed between the 5 MHz and 10 MHz measurements (47 and 40 dB, respectively), raw feature magnitudes and uncorrected regression slopes were not used for inferential comparison of frequency-dependent sensitivity. Statistical analyses were performed using OriginPro 8.5 (OriginLab Corporation, Northampton, MA, USA).

### 2.6. Operational Detection-Index Analysis

The detectability of the submillimeter SUS304 cylindrical surrogate targets was evaluated under the 10 MHz acquisition condition using a study-specific detection index (*DI*). The analysis used the R1 background region and the peak height (*PH*) obtained from the baseline-subtracted difference waveform, as defined in Section 2.4.

The background reference level was calculated as

$$L_{BG}=\mu_{BG}+6\sigma_{BG}$$

where $\mu_{BG}$ and $\sigma_{BG}$ are the mean and standard deviation, respectively, of the absolute difference-signal amplitudes in R1. The dimensionless detection index was calculated as

$$DI=\frac{PH}{L_{BG}}=\frac{PH}{\mu_{BG}+6\sigma_{BG}}$$

A target was considered to meet the operational detection criterion when *DI* > 3. Thus, the criterion required the target-associated peak amplitude to satisfy

$$PH>3\left(\mu_{BG}+6\sigma_{BG}\right)$$

The detection index is a study-specific normalized metric and is not equivalent to a conventional signal-to-noise ratio. The term μ_BG_ + 6σ_BG_ was used as a conservative reference level encompassing background fluctuations, while the additional requirement of DI > 3 required the target-associated peak to exceed three times this reference level. The DI > 3 criterion was specified before comparison of the diameter-dependent results. This combined criterion was used to reduce ambiguous classification of small background fluctuations as target-associated signals. The criterion was used only to identify the smallest tested SUS304 wire target meeting the operational criterion under the present 10 MHz condition and was not intended to represent a validated clinical limit of detection for biological thrombi. Because each stored RF waveform was obtained by internally averaging 256 consecutive acquisitions, the calculated detection index represents the averaged waveform rather than an individual acquisition.

## 3. Results

Representative ultrasonic RF signals were acquired during horizontal scanning of the surrogate targets using the experimental system. A distinct reduction in the transmitted ultrasonic signal was observed when the surrogate target passed through the focal region of the ultrasonic beam. Following baseline removal, the processed difference signals provided substantially improved visualization of the target signal changes compared with the raw RF signals. The corresponding squared-amplitude-sum profiles as a function of scan position clearly identified the target location within the ultrasonic focal region (Figure 3 and Figure 4). Ultrasonic measurements were subsequently performed using surrogate targets of different materials (ABS, acrylic, and SUS304 wire) and diameters. The relationships between target diameter and the extracted ultrasonic signal features are presented in Figure 5. Although the absolute signal amplitudes varied among the target materials because of their different acoustic properties, similar trends were observed for all materials.

Exploratory regression analysis demonstrated positive associations between measured target diameter and all three signal features. Pulse area showed strong linear associations across the six material–frequency conditions, with R^2^ values ranging from 0.904 to 0.997. The squared-amplitude sum showed similarly strong associations, with R^2^ values ranging from 0.898 to 0.997.

The relationship between peak height and diameter varied more substantially among conditions. Linear goodness of fit was lower for 5 MHz ABS (R^2^ = 0.571) and 5 MHz acrylic (R^2^ = 0.741), whereas the remaining peak-height datasets yielded R^2^ values ranging from 0.915 to 0.995. Visual flattening was observed at larger diameters in some peak-height datasets; however, no nonlinear or saturation model was established. Complete regression and correlation results are presented in Table 3.

No single feature consistently yielded the highest R^2^ across all six conditions. Pulse area, peak height, and the squared-amplitude sum each provided the highest R^2^ in two conditions. Pulse area was therefore considered a practical candidate because it maintained strong diameter-dependent associations across all evaluated conditions and was computationally simpler than the squared-amplitude measure, rather than because it demonstrated statistical superiority.

To evaluate operational detectability at submillimeter diameters, the 10 MHz measurements obtained using SUS304 wire targets were analyzed using the detection index defined in Section 2.6 (Table 2). For the 0.1 mm target, a distinct target-associated peak could not be reliably quantified and the result was therefore recorded as NQ. The 0.2 mm target yielded a DI of 1.28 and did not meet the DI > 3 criterion, whereas the 0.3 mm target yielded a DI of 3.94 and met the criterion. Accordingly, the 0.3 mm SUS304 wire target was the smallest tested surrogate target that met the study-specific operational criterion under the specified 10 MHz experimental conditions. This result does not represent a biological thrombus detection limit.

## 4. Discussion

Thrombus formation remains one of the major complications during extracorporeal membrane oxygenation (ECMO) despite continuous advances in circuit technology and anticoagulation strategies. Circuit thrombosis may impair oxygenator performance, increase transmembrane pressure, and eventually result in thromboembolic complications if detached thrombi enter the systemic circulation.

Current surveillance methods rely primarily on indirect indicators, including pressure monitoring, oxygenator performance, laboratory parameters, and visual inspection, all of which have limited capability for detecting individual thrombi or estimating thrombus burden in real time. Furthermore, ECMO itself induces a complex inflammatory and prothrombotic response through continuous blood contact with artificial surfaces, making reliable thrombus monitoring particularly challenging [1,2,27].

Various sensing approaches have therefore been investigated for thrombus monitoring in extracorporeal circulation. Acoustic monitoring using vibration or infrasound analysis and optical sensing based on light-scattering principles have demonstrated the ability to detect thrombus-related changes, while ultrasonic and imaging-based studies have further shown that thrombus formation and coagulation are associated with measurable changes in acoustic or structural characteristics [11,12,17,18,19]. However, these approaches have primarily focused on detecting thrombus formation or the presence of thrombi rather than characterizing how measured signals vary with the size of individual targets. Recent reviews have likewise emphasized the absence of practical technologies capable of continuously monitoring thrombus burden in real time [6].

Our previous study demonstrated that through-transmission ultrasound could detect individual thrombi flowing through an ECMO circuit using noninvasive ultrasonic sensors positioned outside the bloodline [23]. In that study, thrombi measuring approximately 0.5 × 1.0 cm produced reproducible ultrasonic signal changes. However, because only a single thrombus size was evaluated, the relationship between ultrasonic signal characteristics and thrombus size could not be systematically established.

The present study builds upon these observations by using standardized cylindrical surrogate targets to characterize the relationship between target diameter and through-transmission ultrasonic signal features under controlled experimental conditions. The principal finding was that the measured ultrasonic features showed diameter-dependent associations across the evaluated surrogate targets, although the strength and pattern of these associations differed among the evaluated features. This extends our previous work from detection of a single-sized thrombus to controlled characterization of size-dependent ultrasonic responses using standardized targets, without implying that quantitative thrombus sizing has yet been established. Representative previously reported approaches for thrombus monitoring in extracorporeal circuits are summarized in Table 4.

Pulse area showed consistently strong diameter-dependent associations across the evaluated material–frequency conditions. Peak height showed visual flattening at larger diameters in some datasets, particularly for 5 MHz ABS, whereas the squared-amplitude sum showed associations comparable to those of pulse area. No feature demonstrated universally superior goodness of fit. Nevertheless, because pulse area integrates absolute signal deviations over the pulse window without squaring the amplitudes, it may represent a computationally practical candidate for future investigation. This interpretation remains exploratory because independent replicate measurements and predictive-model validation were not performed. Pulse area may be particularly useful for subsequent development because it incorporates changes distributed across the complete difference pulse, is insensitive to signal polarity, and is computationally simpler than the squared-amplitude sum.

Some datasets yielded Spearman’s ρ values of 1.000. However, these values indicate complete rank ordering within the limited tested diameter levels and should not be interpreted as evidence of perfect measurement reliability, repeatability, or predictive accuracy, particularly because independent replicate measurements were unavailable.

Strong positive diameter-dependent associations were observed at both 5 and 10 MHz. However, receiver gain differed between the two conditions (47 dB at 5 MHz and 40 dB at 10 MHz), and the system responses were not calibrated for cross-frequency comparison. Consequently, the absolute feature magnitudes, dynamic ranges, and uncorrected regression slopes cannot establish that either frequency provided superior sensitivity or sizing performance. The present results therefore demonstrate diameter-dependent associations at two operating frequencies rather than comparative frequency superiority.

Under the specified 10 MHz condition, the 0.3 mm SUS304 wire target was the smallest tested surrogate target meeting the study-specific operational detection-index criterion. Because SUS304 has substantially greater acoustic impedance contrast relative to water than biological thrombi relative to blood, this result should not be interpreted as demonstrating detection of a biological thrombus of the same size or as a clinically applicable detection limit.

The present findings extend qualitative target detection by demonstrating diameter-dependent associations between target diameter and through-transmission ultrasonic signal features under controlled experimental conditions. Quantitative size estimation will require the development and validation of a predictive sizing model in future studies. In particular, a clinically applicable sizing approach would require independently repeated measurements, assessment of measurement variability and prediction error, and validation using biological thrombi across a range of sizes and compositions.

The ability to estimate thrombus size could potentially provide information beyond simple thrombus detection. However, the present study did not evaluate biological thrombus sizing, thrombus burden, or clinical outcomes, and therefore no direct clinical inference can be made from the current target measurements. If validated in subsequent biological and ECMO-circuit studies, size-dependent ultrasonic information may ultimately complement existing surveillance parameters used during ECMO [2,6,9,28,29,30].

Several limitations should be acknowledged.

First, the present system employed point-focused ultrasonic transducers with a nominal focal length of 2 inches. Scanning a 1 mm-diameter cylindrical target yielded an effective lateral interaction width of less than 2 mm; however, the acoustic focal field was not independently profiled. Therefore, the effective spatial resolution was not experimentally established. Importantly, detection of a 0.3 mm SUS304 wire target should not be interpreted as a spatial resolution of 0.3 mm, because a target smaller than the effective beam width may still produce a measurable change in the transmitted signal.

In addition, the point-focused configuration interrogates only a limited region of the ECMO bloodline and cannot simultaneously monitor the entire lumen. Consequently, thrombi passing outside the effective focal region may be missed by the present configuration. Future systems should therefore investigate array-based or wider-beam ultrasonic configurations capable of increasing spatial coverage.

Second, surrogate materials including acrylic, ABS, and stainless steel were used instead of biological thrombi. Although the reported acoustic impedances of acrylic and ABS are numerically closer to those of blood and biological clot than that of stainless steel, similarity in acoustic impedance alone does not establish comprehensive acoustic equivalence to biological thrombi. The measured through-transmission response may also depend on sound speed, attenuation, interfacial reflection, scattering, and surface characteristics. Because these properties were not independently characterized in the present study, the differences in absolute signal magnitude among the materials cannot be attributed solely to acoustic impedance. Accordingly, the present analysis emphasizes diameter-dependent trends within each material rather than direct quantitative comparisons among materials. The observed responses should not be directly extrapolated to biological thrombus detection or sizing.

Nevertheless, our previous study demonstrated measurable ultrasonic signal changes produced by real thrombi within ECMO circuits, supporting the feasibility of the present experimental approach [23]. The current study should therefore be viewed as a controlled intermediate step between our previous demonstration of biological thrombus detection and the future development of quantitative thrombus-sizing approaches, rather than as validation of quantitative thrombus sizing itself. Third, although each RF waveform was obtained by internal averaging of 256 consecutive acquisitions, independent repeated measurements were not performed. Therefore, measurement variability and reproducibility could not be quantitatively assessed in the present study. Future studies should incorporate independent repeated measurements to evaluate the variability and reproducibility of the extracted ultrasonic signal features.

Although the same symmetric spatial baseline-selection rule was used for all measurements and no evident baseline drift was observed, the effects of alternative baseline-region widths or position-selection rules were not quantitatively evaluated. Because the extracted features were calculated from baseline-subtracted waveforms, variation in baseline selection could influence their absolute values. Future studies should prospectively define and automate the baseline-selection procedure and evaluate its sensitivity and reproducibility.

Finally, only cylindrical surrogate targets were evaluated. All targets were at least 50 mm long, and their central portions were insonified sufficiently far from both ends; therefore, end effects were expected to be small relative to the focal interaction region. The target longitudinal axis was aligned perpendicular to the ultrasound propagation direction within an estimated tolerance of 1°. For an ideal cylindrical geometry, this tolerance corresponds to a central geometric path-length change of approximately 0.015%; nevertheless, residual effects of alignment and surface geometry cannot be excluded.

Nevertheless, the fixed cylindrical geometry and orientation used in this study do not reproduce the irregular morphologies and variable orientations of biological thrombi in clinical ECMO circuits. Such variations may alter the projected cross-sectional area and effective acoustic path length and consequently affect the transmitted ultrasonic signal. Future studies should therefore evaluate biological thrombi with variable shapes and orientations.

In clinical ECMO circuits, thrombi also exhibit continuously changing compositions as fibrin organization, platelet aggregation, and erythrocyte incorporation evolve during thrombus maturation. These structural changes are known to alter the acoustic properties of thrombi and may influence ultrasonic signal characteristics [17,18,19,20,21,22].

In addition, the present water-chamber model does not reproduce the effects of blood, tubing walls, blood flow, hematocrit, or red-cell aggregation on ultrasonic transmission. Therefore, the present findings should be interpreted as controlled acoustic target measurements rather than as a direct simulation of ECMO thrombus monitoring.

Future studies should therefore investigate biologically generated thrombi with realistic morphologies and variable orientations under physiological ECMO flow conditions and include independent repeated measurements across a broader range of thrombus sizes and ultrasonic frequencies. Such studies will be required to develop and validate predictive algorithms that account for thrombus geometry and acoustic properties for quantitative thrombus sizing.

## 5. Conclusions

This study demonstrated diameter-dependent changes in through-transmission ultrasonic signals obtained from standardized cylindrical surrogate targets under controlled conditions. Pulse area was identified as a practical candidate feature because of its consistently strong diameter-dependent associations and computational simplicity. However, the exploratory analysis did not establish its statistical superiority or validate a predictive sizing model.

Under the specified 10 MHz condition, the 0.3 mm SUS304 wire target was the smallest tested surrogate target meeting the study-specific operational detection-index criterion. This result does not represent a biological thrombus detection limit. Validation using biological thrombi under physiologically representative ECMO flow conditions is required before quantitative thrombus sizing can be established.

## Figures and Tables

**Figure 1:**
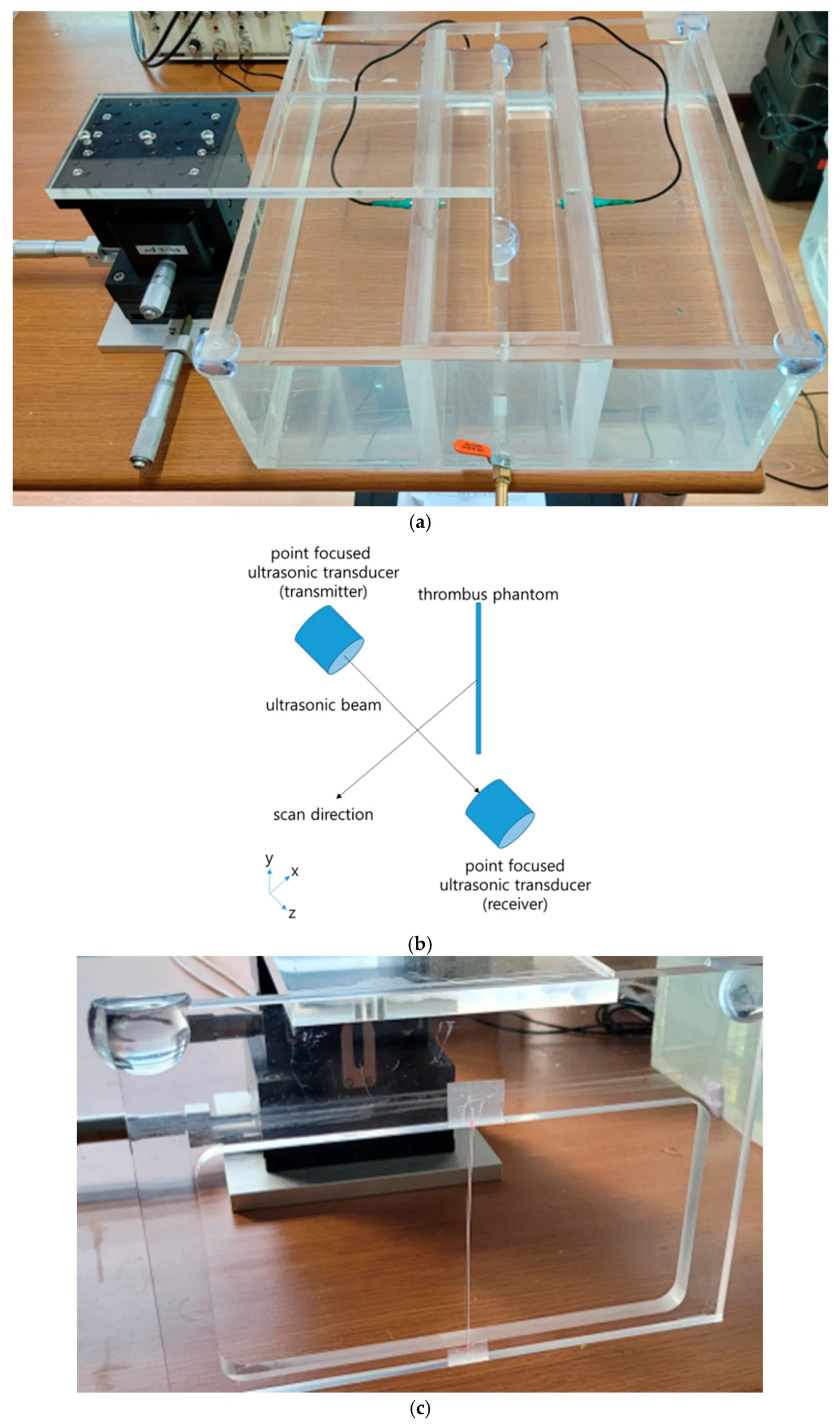
Experimental setup and measurement principle for ultrasonic surrogate target characterization. (**a**) Photograph of the experimental water chamber used for ultrasonic measurements. (**b**) Schematic illustration of the ultrasonic measurement principle showing ultrasonic wave propagation through the surrogate target. (**c**) Photograph of the cylindrical surrogate target mounted vertically on the manual translation stage for controlled positioning during ultrasonic measurements. The cylindrical surrogate target was positioned between the transmitting and receiving transducers, with its longitudinal axis perpendicular to the ultrasound propagation direction within 1°. The central portion of the target was located in the focal region. All targets were at least 50 mm long, whereas scanning a 1 mm-diameter cylindrical target yielded an effective lateral interaction width of less than 2 mm.

**Figure 2:**
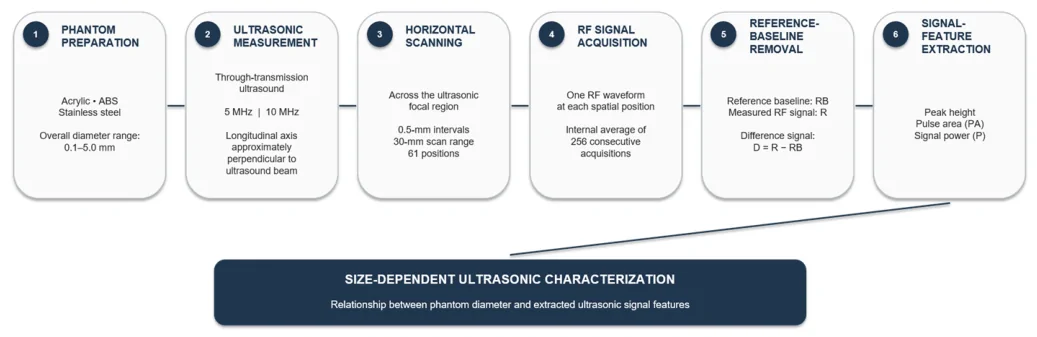
Experimental workflow for size-dependent ultrasonic characterization of cylindrical surrogate targets. Standardized cylindrical surrogate targets were scanned horizontally across the through-transmission ultrasonic beam using 5 or 10 MHz transducers. RF waveforms were acquired at 0.5 mm intervals over a 30 mm scan range, followed by reference baseline removal and extraction of peak height, pulse area, and squared-amplitude sum. The relationships between the surrogate target diameter and the extracted ultrasonic signal features were subsequently characterized under the 5 and 10 MHz conditions.

**Figure 3:**
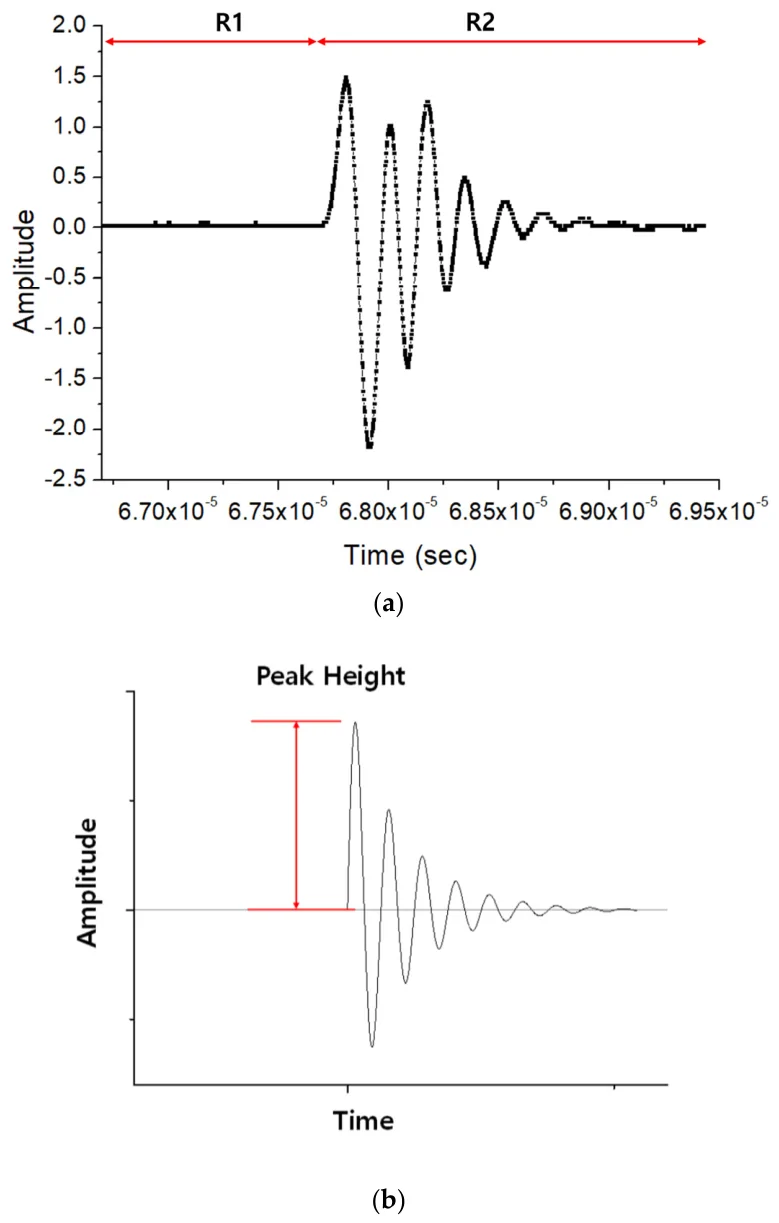

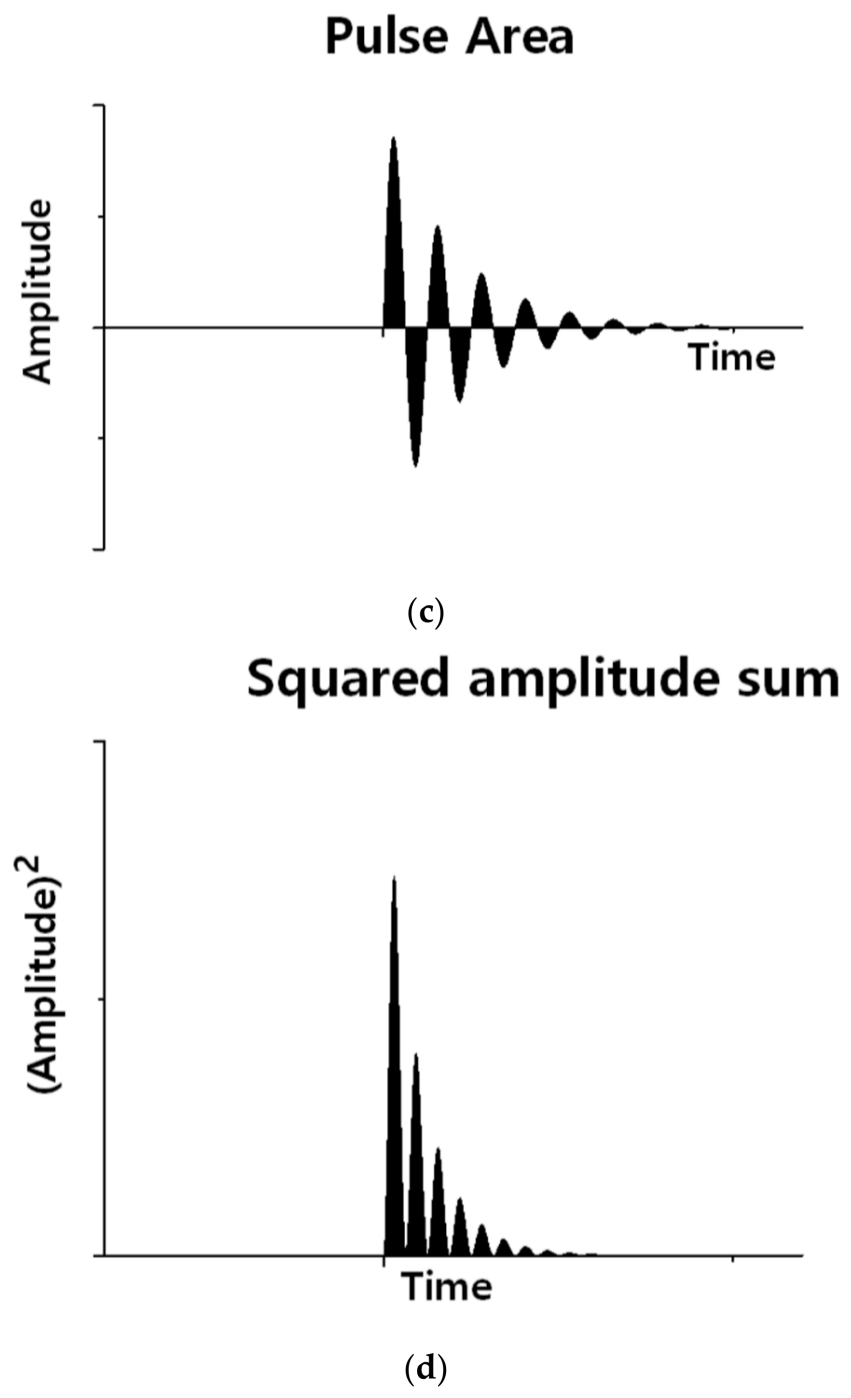
Definition of the signal-analysis regions and extracted features. (**a**) Representative ultrasonic RF waveform acquired during the experiment. The raw RF waveform was initially aligned so that its first absolute 50 mV threshold crossing occurred at approximately 40% of the 2.5 μs acquisition window. After reference baseline subtraction, the first point at which the absolute difference-signal amplitude exceeded 50 mV defined the boundary between R1 and R2. R1 represents the pre-pulse background region used to estimate the background noise level, whereas R2 contains the target-associated difference pulse used for feature extraction. (**b**) Peak height extracted from R2. (**c**) Pulse area extracted from R2. (**d**) Squared-amplitude sum (P) extracted from R2. The 50 mV threshold used to define the signal-analysis regions was distinct from the DI-based operational detection criterion.

**Figure 4:**
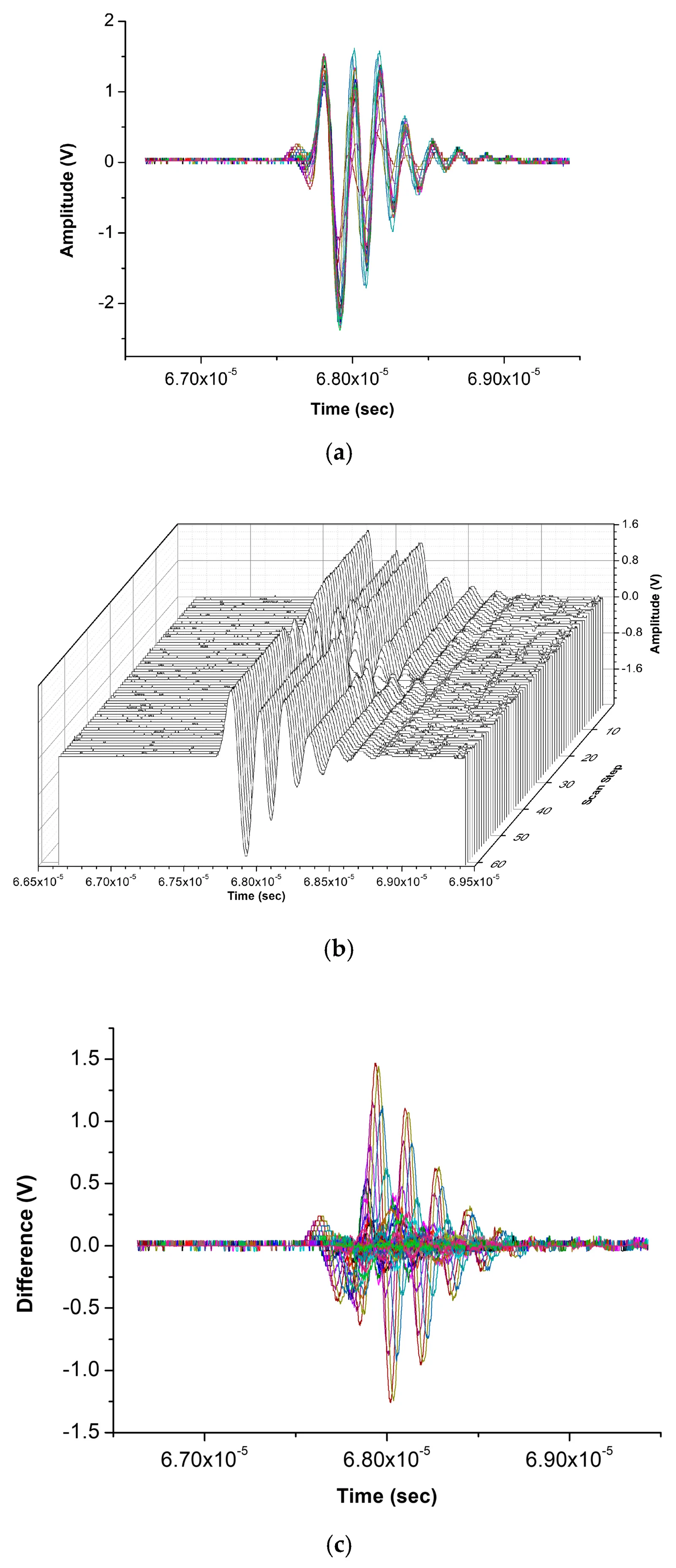

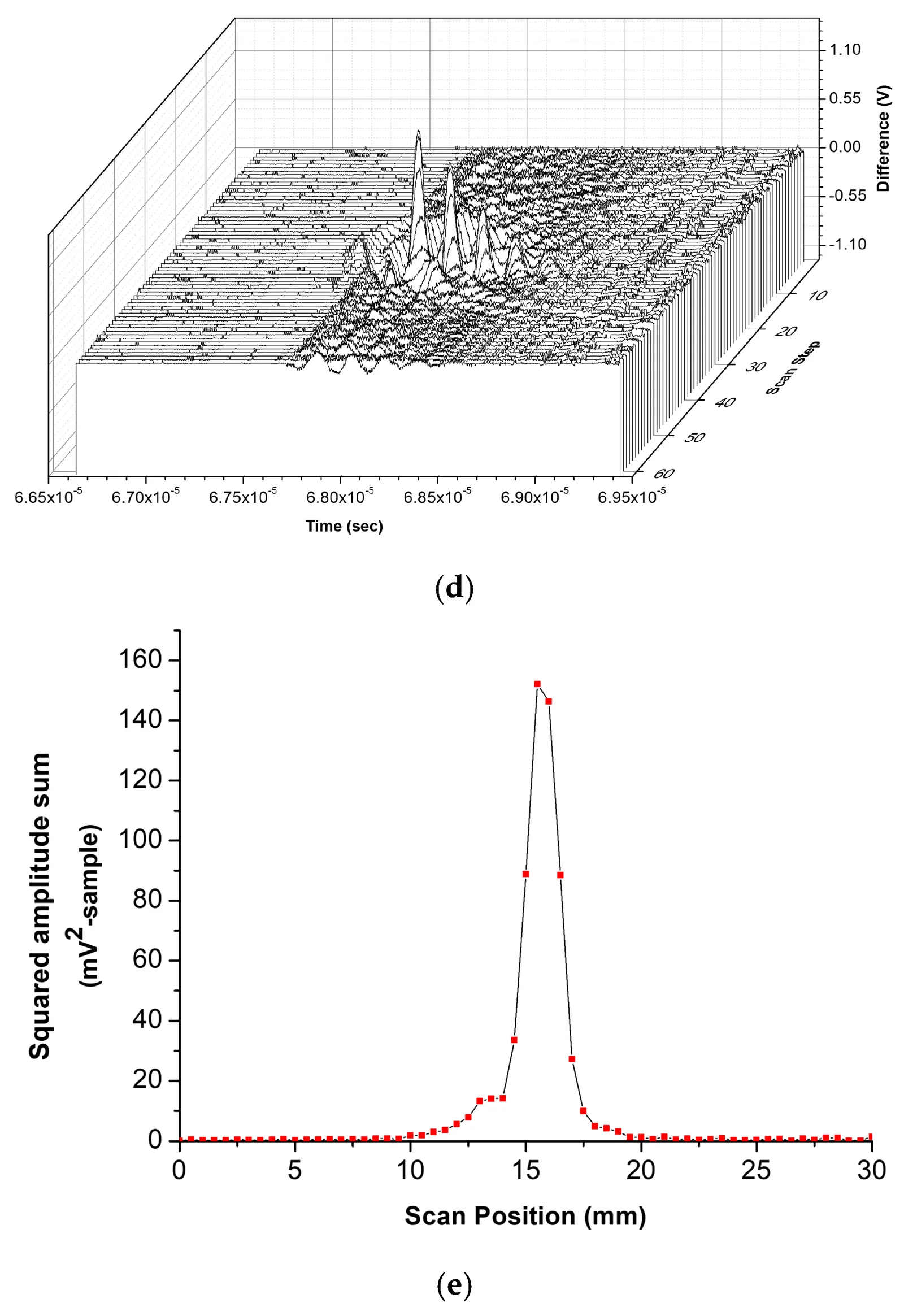
Signal-processing procedure for ultrasonic surrogate target characterization. (**a**) Raw ultrasonic RF waveforms acquired during horizontal scanning of a 1.0 mm ABS target at 61 positions. (**b**) Three-dimensional representation of the raw waveforms. (**c**) Difference waveforms obtained after target-specific spatial-reference baseline subtraction. (**d**) Three-dimensional representation of the difference waveforms. (**e**) Squared-amplitude sum as a function of scan position, showing localization of the target within the focal region.

**Figure 5:**
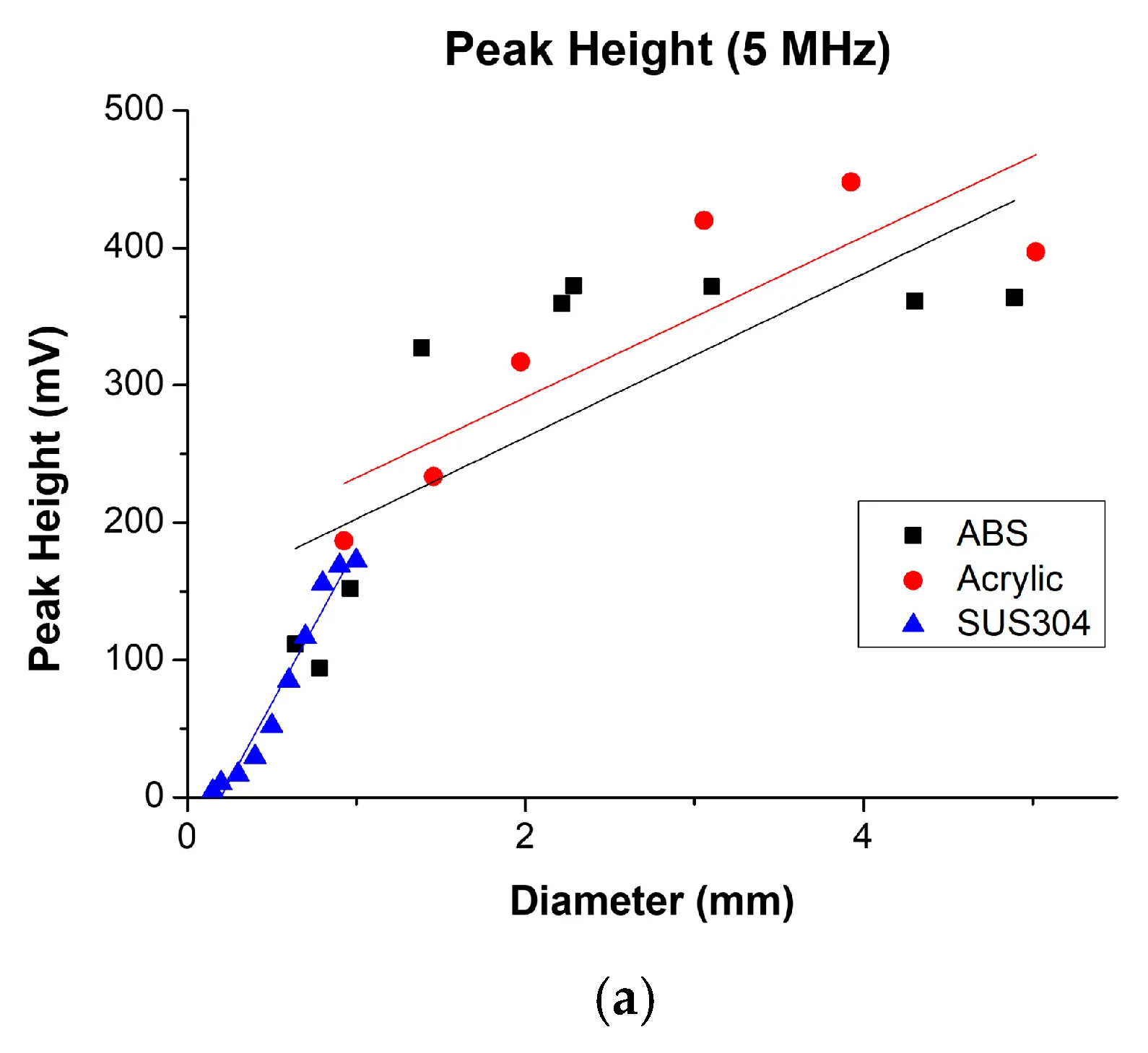

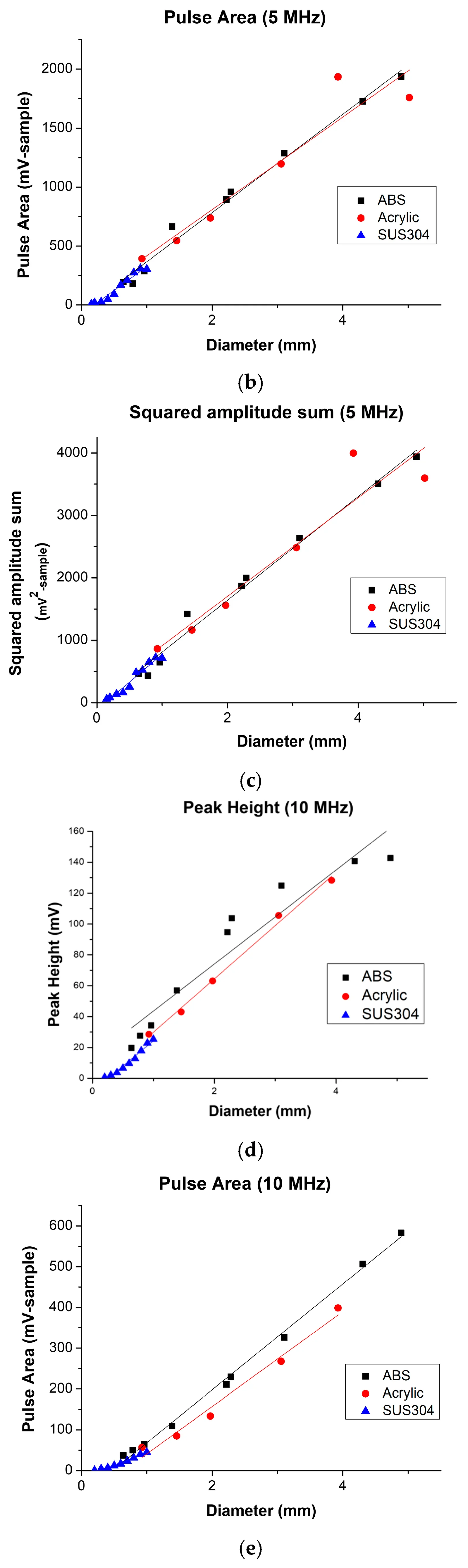

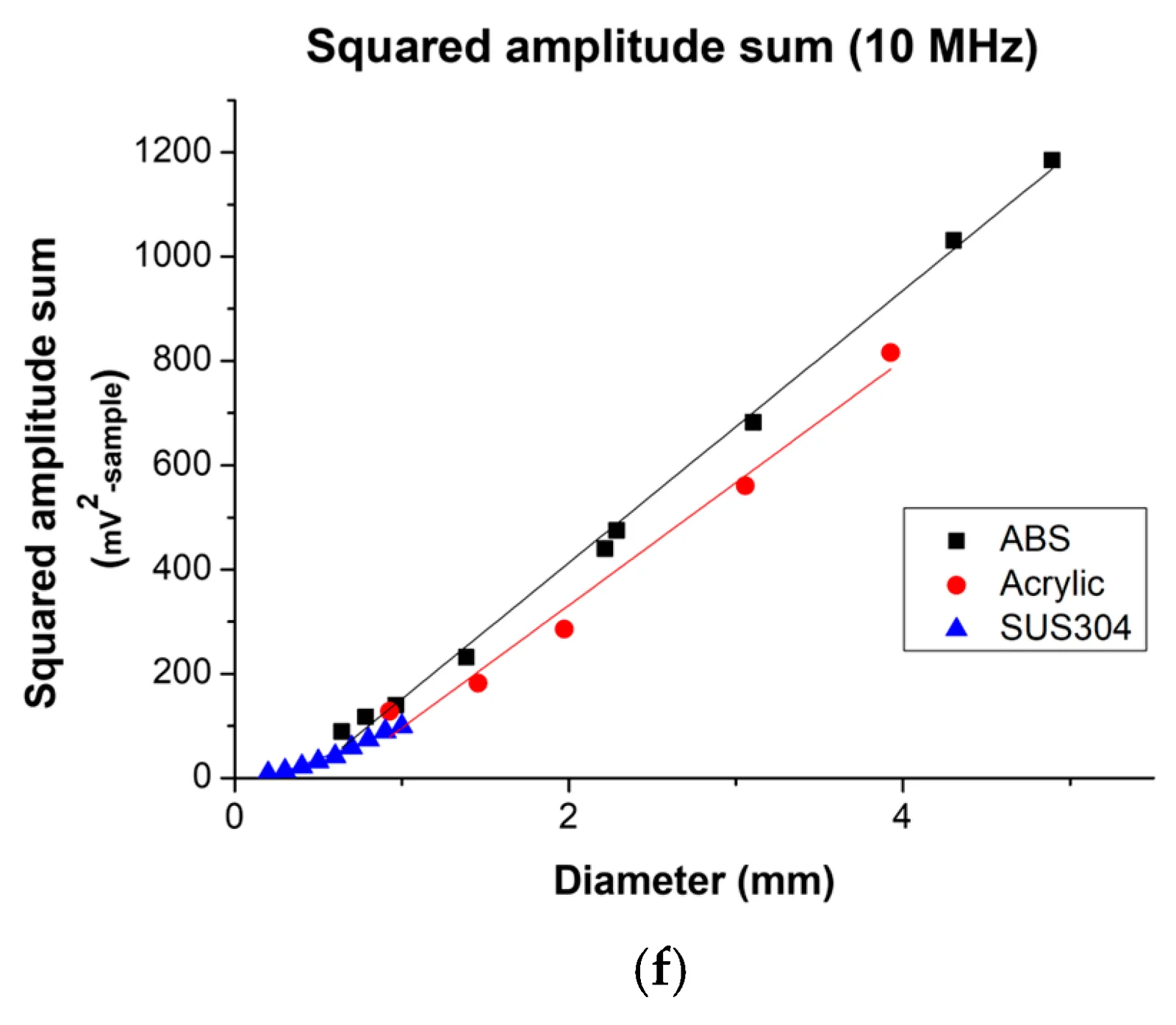
Relationships between measured target diameter and ultrasonic signal features for cylindrical surrogate targets. Panels (**a**–**c**) show peak height, pulse area, and squared-amplitude sum, respectively, at 5 MHz; panels (**d**–**f**) show the corresponding features at 10 MHz. Black squares, red circles, and blue triangles represent ABS, acrylic, and SUS304 wire targets, respectively. Each marker represents the single measured value obtained at the corresponding target diameter. Solid lines represent material-specific exploratory linear regression fits and do not connect consecutive measurements. No error bars are shown because independent replicate measurements were unavailable at each diameter. Regression and correlation results are reported in Table 3. Receiver gain was 47 dB at 5 MHz and 40 dB at 10 MHz; therefore, absolute feature magnitudes and dynamic ranges should not be interpreted as direct measures of relative frequency sensitivity.

**Table 1:** Materials, shapes, and dimensions of the surrogate targets.

Material	Shape	Dimensions
Acrylic	Cylinder	Diameter: 1.0, 1.5, 2.0, 3.0, 4.0 and 5.0 mm; length ≥ 50 mm
ABS	Cylinder	Diameter 0.5, 0.8, 1.0, 1.5, 2.0, 2.5, 3.0, 4.0, and 5.0 mm; length ≥ 50 mm
SUS304 stainless steel	Wire	Diameter: 0.1–1.0 mm (0.1 mm increments); length ≥ 50 mm

**Table 2:** Detection indices of submillimeter SUS304 wire targets measured under the 10 MHz acquisition condition.

SUS304 Wire Diameter (mm)	Background Mean, μ_BG_ (V)	Background SD, σ_BG_ (V)	Background Reference Level, L_BG_ = μ_BG_ + 6σ_BG_ (V)	Peak Height, PH(V)	Detection Index, DI = PH/L_BG_
0.1	0.132	0.122	0.867	NQ	NQ
0.2	0.113	0.075	0.561	0.72	1.28
0.3	0.094	0.070	0.513	2.02	3.94
0.4	0.136	0.112	0.810	3.65	4.50
0.5	0.129	0.097	0.710	6.63	9.34
0.6	0.123	0.100	0.723	9.77	13.51
0.7	0.178	0.199	1.371	12.78	9.32
0.8	0.183	0.167	1.186	17.89	15.08
0.9	0.114	0.089	0.650	22.85	35.15
1.0	0.124	0.082	0.619	25.34	40.94

Note: Measurements were obtained using the paired 10 MHz point-focused transducers with a pulser output of 215 V, a receiver gain of 40 dB, a sampling rate of 500 MS/s, a pulse repetition frequency of 1 kHz, no damping, a 1 MHz high-pass filter, no low-pass filter, and internal averaging of 256 consecutive acquisitions. The background reference level was calculated as LBG = μBG + 6σBG using absolute difference-signal amplitudes in R1. The detection index was calculated as DI = PH/LBG, where PH was the maximum absolute difference-signal amplitude in R2. DI > 3 was used as the study-specific operational criterion. NQ indicates that a distinct target-associated peak could not be reliably quantified. The reported DI characterizes the internally averaged waveform rather than an individual acquisition.

**Table 3:** Exploratory regression and correlation analyses.

Spearman ρ	*p*	R^2^	Slope (95% CI)	*n*	Condition
0.833	0.0186	0.571	59.5 (13.4–105.7)	9	5 MHz/ABS/PH
0.983	5.97 × 10^−8^	0.988	417.0 (375.5–458.6)	9	5 MHz/ABS/PA
0.983	8.07 × 10^−8^	0.987	830.4 (744.1–916.8)	9	5 MHz/ABS/P
0.829	0.0276	0.741	58.5 (10.5–106.5)	6	5 MHz/Acrylic/PH
0.943	0.00354	0.904	392.2 (215.2–569.3)	6	5 MHz/Acrylic/PA
0.943	0.00405	0.898	789.7 (420.2–1159.3)	6	5 MHz/Acrylic/P
1.000	4.72 × 10^−7^	0.964	225.7 (190.1–261.3)	10	5 MHz/SUS304/PH
0.988	6.98 × 10^−7^	0.960	405.3 (338.0–472.6)	10	5 MHz/SUS304/PA
0.988	7.62 × 10^−7^	0.959	892.2 (742.4–1042.0)	10	5 MHz/SUS304/P
1.000	5.44 × 10^−5^	0.915	30.5 (22.2–38.8)	9	10 MHz/ABS/PH
1.000	6.83 × 10^−10^	0.997	129.8 (123.0–136.6)	9	10 MHz/ABS/PA
1.000	4.13 × 10^−10^	0.997	261.2 (248.5–273.9)	9	10 MHz/ABS/P
1.000	0.000158	0.995	34.5 (30.0–39.1)	5	10 MHz/Acrylic/PH
1.000	0.00110	0.981	116.3 (86.7–145.8)	5	10 MHz/Acrylic/PA
1.000	0.000975	0.983	235.2 (177.8–292.6)	5	10 MHz/Acrylic/P
1.000	1.24 × 10^−6^	0.971	32.6 (27.5–37.6)	9	10 MHz/SUS304/PH
1.000	6.15 × 10^−7^	0.976	57.7 (49.7–65.8)	9	10 MHz/SUS304/PA
1.000	3.69 × 10^−7^	0.979	120.3 (104.7–135.9)	9	10 MHz/SUS304/P

Note: Slopes were estimated using measured target diameter. The 95% confidence intervals describe uncertainty in the fitted regression slopes and do not quantify within-diameter repeatability because only a single measured value was available at each diameter. Analyses were exploratory. Raw slopes should not be compared across signal features because the features have different dimensions, or between frequencies because receiver gain differed.

**Table 4:** Comparison of representative approaches for thrombus monitoring in extracorporeal circuits.

Study	Monitoring Approach	Sensing Principle	Study Setting	Key Feature
Fuchs et al. [11]	Infrasound monitoring	Low-frequency acoustic signals (<20 Hz)	Clinical ECMO and laboratory experiments	Detection of circulating thrombotic material based on changes in the acoustic spectrum
Morita et al. [12]	Micro-optical sensing	Light scattering at 660 and 855 nm	In vitro extracorporeal circuit with porcine blood	Real-time detection of thrombus formation using optical sensors
Slapal et al. [14]	Bioimpedance monitoring	Electrical impedance measurement with machine-learning analysis	Computational ECMO oxygenator model	Detection of thrombus-associated impedance changes
Rim et al. [23]	Through-transmission ultrasound	Changes in transmitted ultrasonic signals	Modified in vitro ECMO circuit with porcine blood	Real-time detection of circulating thrombi using externally positioned ultrasonic sensors
Present study	Through-transmission ultrasound	Size-dependent changes in transmitted ultrasonic signal features	Controlled experiments with standardized cylindrical surrogate targets	Characterization of relationships between target diameter and ultrasonic signal features

## Data Availability

The data presented in this study are available on request from the corresponding author due to ongoing research purpose.
